# Completely ES Cell-Derived Mice Produced by Tetraploid Complementation Using Inner Cell Mass (ICM) Deficient Blastocysts

**DOI:** 10.1371/journal.pone.0094730

**Published:** 2014-04-14

**Authors:** Duancheng Wen, Nestor Saiz, Zev Rosenwaks, Anna-Katerina Hadjantonakis, Shahin Rafii

**Affiliations:** 1 Ansary Stem Cell Institute and Department of Genetic Medicine, Weill Cornell Medical College, New York, New York, United States of America; 2 Ronald O. Perelman and Claudia Cohen Center for Reproductive Medicine, Weill Cornell Medical College, New York, New York, United States of America; 3 Developmental Biology Program, Sloan Kettering Institute, New York, New York, United States of America; Institute of Zoology, Chinese Academy of Sciences, China

## Abstract

Tetraploid complementation is often used to produce mice from embryonic stem cells (ESCs) by injection of diploid (2n) ESCs into tetraploid (4n) blastocysts (ESC-derived mice). This method has also been adapted to mouse cloning and the derivation of mice from induced pluripotent stem (iPS) cells. However, the underlying mechanism(s) of the tetraploid complementation remains largely unclear. Whether this approach can give rise to completely ES cell-derived mice is an open question, and has not yet been unambiguously proven. Here, we show that mouse tetraploid blastocysts can be classified into two groups, according to the presence or absence of an inner cell mass (ICM). We designate these as type *a* (presence of ICM at blastocyst stage) or type *b* (absence of ICM). ESC lines were readily derived from type *a* blastocysts, suggesting that these embryos retain a pluripotent epiblast compartment; whereas the type *b* blastocysts possessed very low potential to give rise to ESC lines, suggesting that they had lost the pluripotent epiblast. When the type *a* blastocysts were used for tetraploid complementation, some of the resulting mice were found to be 2n/4n chimeric; whereas when type *b* blastocysts were used as hosts, the resulting mice are all completely ES cell-derived, with the newborn pups displaying a high frequency of abdominal hernias. Our results demonstrate that completely ES cell-derived mice can be produced using ICM-deficient 4n blastocysts, and provide evidence that the exclusion of tetraploid cells from the fetus in 2n/4n chimeras can largely be attributed to the formation of ICM-deficient blastocysts.

## Introduction

Mouse diploid (2n) embryos can be induced to become tetraploid (4n) by blastomere fusion at the 2-cell stage or by temporary inhibition of embryonic mitosis at the 1-cell stage. The resulting tetraploid embryos have a delay in one round of cell division and thus have fewer cells than age-matched diploid embryos. Interestingly 4n embryos undergo compaction and blastocyst cavity formation at equivalent times as diploid embryos [1]. Tetraploid embryos can develop to the blastocyst stage *in vitro*, and depending on strain background, can survive at very low frequencies up to 14 and 15 days post coitum (dpc) *in vivo* [2], [3], [4], [5]. When mouse tetraploid embryos are combined with diploid cells in chimeras, the tetraploid cells usually exhibit a restricted tissue distribution in the resulting embryos, with tetraploid cells usually excluded from the epiblast lineage at early post-implantation stages[5], [6], [7], [8], [9], [10], [11]. This distinctive bias in the lineage potency of 4n embryos and 2n ES cells has been applied to generate completely ES cell-derived mice by aggregating 2n ES cells with 4n embryos or by injecting diploid ES cells into 4n blastocysts. The 4n compartment will complement the developmental potential of ES cells, which are restricted to forming only epiblast derivatives, and thus supply the derivatives of the trophectoderm and primitive endoderm compartments of the blastocyst[12], [13], and this process is often referred to as tetraploid complementation [8], [11], [14].

This method has been adapted to mouse somatic cell nuclear transfer where it is referred to as the two-step cloning method [15], [16], [17], and lately it has been used to derive adult mice from induced pluripotent stem cells (iPS) [18], [19]. Notably, the underlying mechanism(s) of the tetraploid complementation remain largely unknown. Furthermore, whether this approach can give rise to completely ES cell-derived mice is still debated [20]; since often mice produced by tetraploid complementation were found to be 2n/4n chimeras [11], [20], [21].

Here, we show that mouse tetraploid blastocysts usually fall into two groups, as judged by the presence or absence of an ICM, designated type *a* (presence of ICM) or type *b* (absence of ICM). Type *b* blastocysts lack an OCT4+ ICM and are unable to give rise to ESC lines, whereas type *a* blastocysts do so at similar frequencies than 2n blastocysts. We demonstrate that both type *a* and type *b* blastocysts exhibit similar potential to produce mice when injected with diploid ESCs. However, mice derived from type *a* blastocysts were frequently found to be diploid/tetraploid (2n/4n) chimeras after birth, whereas mice derived from the ICM-deficient, type *b* blastocysts are completely ES cell-derived. Our results thus provide further insight into the mechanism of tetraploid complementation and establish a tool for a more efficient generation of all-ESC derived mice.

## Materials and Methods

### Mice and embryos

Animals were housed and prepared according to the protocol approved by the IACUC of Weill Cornell Medical College (Protocol number: 2009-0061). Wild-type mice were purchased from Taconic Farms (Germantown, NY) and The Jackson Laboratory (Bar Harbor, ME). Mice of strain *Tg(Pou5f1-EGFP)2Mnn/J* (abbreviated *Oct4-*EGFP) were purchased from The Jackson Laboratory. Females were superovulated at 6–8 weeks with 5 IU PMSG (Pregnant mare serum gonadotrophin, Sigma-Aldrich, St. Louis, MO) and 5 IU hCG (Human chorionic gonadotrophin, Sigma-Aldrich) at intervals of 48 h. The females were mated individually to males, and checked for the presence of a vaginal plug the following morning. Plugged females were sacrificed by cervical dislocation at 1.5 days after hCG injection for the collection of 2-cell embryos. These embryos were flushed from the oviducts with KSOM+AA (Specialty Media), and cultured in KSOM for 2.5 days in vitro at 37°C under 5% CO_2_ in air to the blastocyst stage.

Embryos at the 2-cell stage were subjected to electrofusion for induction of tetraploidy. Embryos were washed in 0.3M d-mannitol (Sigma-Aldrich) and 0.3% BSA (Sigma-Aldrich) for 20 sec and transferred to a fusion chamber attached to an ECM2001 Electrocell Manipulator (BTX Inc., San Diego, CA). Two-cell embryos were aligned so that the plane of intersection of the blastomeres was perpendicular to the electric field using an alternating current and double direct current; pulses of 1,000 V/cm for 30 µs were applied. Following application of the electric field, embryos were transferred to KSOM+AA and cultured in an incubator. Blastomere fusion was checked 2 h after application of the DC electric field; fused embryos were moved to new KSOM+AA micro drops covered with mineral oil, and cultured further in an incubator under 5% CO_2_ at 37°C.

### Derivation and culture of ESC lines

The ES cell derivation medium (ESDM) was slightly modified from the published protocols[22], [23]: 75 ml Knockout DMEM (SR, Gibco, Cat# 10829-018), 20 ml Knockout Serum Replacement (SR, Gibco, Cat# 10828), 1 ml penicillin/streptomycin (Specialty Media, Cat#TMS-AB-2C), 1 ml L-glutamine (Specialty Media, Cat# TMS-001-C), 1 ml Nonessential Amino Acids (Specialty Media, Cat #TMS-001-C), 1 ml Nucleosides for ES cells (Specialty Media, Cat# ES-008-D), 1 ml β-mercaptoethanol (Specialty Media, Cat# ES-007-E), 250 µl PD98059 (Promega product, Cat# V1191) and 20 µl recombinant mouse LIF (Chemicon International, Cat #ESG1107).

Feeder cells of mouse embryonic fibroblasts (MEFs) were obtained from E12.5 or E13.5 mouse embryos and inactivated with mitomycin C (Sigma cat # M4287). Feeder layers were cultured in serum-supplemented medium overnight, and washed with 1X PBS to reduce serum just prior to plating embryos or ES cells. Diploid or tetraploid blastocysts were used for ESC line derivation. Zonae pellucidae of blastocysts were removed by brief exposure to Tyrode's saline acidified to pH 2.5. These denuded embryos were plated individually into individual wells of a 96-well plate that had been seeded with MEF cells, and cultured in ESDM at 37°C in 5% CO_2_ in humidified air for 4–5 days. Cell clumps originated from the blastocysts were trypsinized in 20 µl of 0.025% Trypsin and 0.75 mM EDTA (Specialty Media, Cat# SM-2004-C) for 5 min, and 200 µl of ESDM was added to each well to stop the reaction. Cells were dispersed by pipetting up and down at least 20 times with a 200 µl pipettor, and the entire medium including the cell suspension was transferred to another well containing freshly seeded MEF cells in the 96-well plate. Cell colonies could be observed 2–3 days after the first trypsinization. Colony expansion of putative ES cells proceeded from 48-well plates to 6-well plates with MEF cells in ESDM, and then to gelatinized 25 cm^2^ flasks for routine culture in ESC culture medium, with 15% fetal calf serum FCS and 1,000 IU/ml LIF. Cell aliquots were cryopreserved using Cell Culture Freezing Medium (Specialty Media, Cat# ES-002-D) and stored in liquid nitrogen.

### Immunohistochemistry staining of ESCs and blastocysts

For embryo immunohistochemical staining, embryos were fixed (4% paraformaldehyde), permeabilized (0.5% Triton X-100 in PBS), blocked (10% Normal donkey serum and 0.5% Triton in PBS) and incubated in working dilutions of the following antibodies. As primary antibodies, anti-OCT4 mouse IgG (BD Biosciences, Cat# 611202, 1∶100), anti-CDX2 rabbit IgG (Millipore, Cat # AB4123, 1∶200) were used. As secondary antibodies anti-rabbit, and anti-mouse IgG, conjugated with Alexa Fluor 546 (A-11036) and 647 (A-21245) were applied (all Invitrogen). Imaging was performed with Zeiss 710 confocal imaging system. Z-stack images of 20 consecutive optical sections for each embryo were acquired. Z-stack projections were generated and cell numbers were counted for each embryo as described previously [24].

The ES Cell Marker Sample Kit (Chemicon, Temecula, CA, Cat# SCR002) was used for the assessment of expression of markers that reflect the undifferentiated state of ESCs. Staining procedures were performed according to the instructions provided by the manufacturer. Briefly, cultured ES cells were fixed in 4% paraformaldehyde/PBS for 15–20 min at room temperature. Cells were permeabilized with 0.1% Triton X-100/PBS for 10 min after washing twice (5–10 min each) with 20 mM Tris-HCl, pH 7.4, 0.15 NaCl, 0.05% Tween-20. Blocking solution (4% normal goat serum/PBS) was applied to cells for 30 min, and cells were incubated with the primary antibodies for 1 hr at room temperature. Cells were further incubated with secondary antibodies for 30–60 min and observed with a fluorescence microscope. Alkaline phosphatase staining was performed according to the manufacturer's protocol (Chemicon, Cat# SCR004).

### Flow cytometric analysis and karyotyping

Single cell suspensions of 1–5 million ESCs were washed twice in ice-cold PBS, and re-suspended cells were washed in 200 ul of PBS/0.1% FBS by vortexing or by pipetting with a small tip. Cells were fixed by adding 10 ml of ice-cold 70% ethanol, and incubated for 1 h to overnight at 4°C. After spinning and removing the ethanol, cells were resuspended in 0.5 ml of propidium iodide solution (40 µg/ml PI and 100 µg/ml RNaseA). Cells were incubated at 37°C for 1 h and filtered through 40–70 µm mesh filters prior to analysis.

Chromosomes of ESCs were stained using Giemsa and karyotyping was performed using the Spectral Imaging software (Vista, CA), according to the manufacturer's protocols. More than 50 metaphase nuclei (for Giemsa staining) or 15–20 metaphase nuclei (for karyotyping) were examined for each cell line.

### Blastocyst injection

Diploid blastocysts were collected from the uterus of E3.5 superovulated ICR females. Tetraploid blastocysts were obtained by electrofusion of two-cell embryos from either superovulated B6D2F1 or ICR females crossed with *Oct4*-EGFP males as described above. Type *a* or type *b* tetraploid blastocysts were identified at day 5 post hCG injection under the fluorescence microscope by the presence/absence of a GFP+ ICM. 2n ESCs were injected into tetraploid blastocysts, and 4n ESCs were injected into diploid blastocysts. For blastocyst injection, ES cells were trypsinized, resuspended in DMEM without LIF, and kept on ice. A flat tip microinjection pipette was used for ESC injection. ESCs were picked up in the end of the injection pipette and 10–15 ESCs were injected into each blastocyst. The injection pipette was used to collect ESCs as a clump and to place them close to the ICM of the blastocyst. The injected blastocysts were kept in KSOM + AA until embryo transfer. Ten injected blastocysts were transferred into each uterine horn of 2.5 dpc pseudopregnant ICR females.

### Data analysis

All data are presented as mean ± SD (Standard deviation) or percentage. Differences between groups were tested for statistical significance using Student's *t-*test (mean data) or χ^2^- test (percentage data). Statistical significance was set at *P*<0.01.

## Results

### Mouse tetraploid blastocysts can be classified into two types according to the presence or absence of ICM

**Figure 1:**
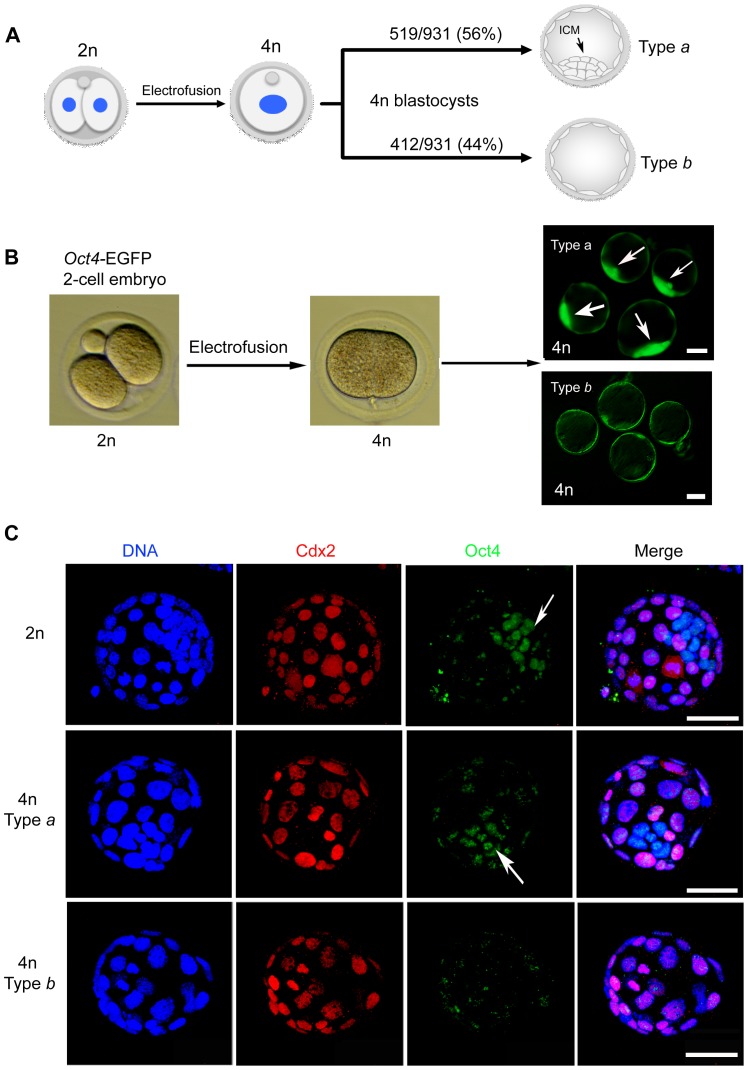
Mouse tetraploid blastocysts are grouped into two types. (A) Schematic illustration of tetraploid embryo generation. The two blastomeres of 2-cell stage diploid (2n) embryos are electrofused into one large blastomere thus doubling the DNA content to tetraploid (4n) in the embryos. The resulting 4n embryos can normally develop to blastocysts and are classified into two groups by the presence (type *a*) or absence (type *b*) of an ICM. (B) The ICM in 4n blastocysts of *Oct4*-EGFP embryos can be visualized by expression of EGFP in the resulting 4n blastocysts, and are classified into type *a* or type *b* under the fluorescence microscope. (C) Confocal images of diploid and tetraploid type *a* and tetraploid type *b* blastocysts, images are full projections of 20 optical sections. Embryos were stained with antibodies of CDX2 (staining the trophoblast) and OCT4 (staining the ICM). Both the diploid and tetraploid type *a* blastocysts showed ICM in the embryos, whereas the tetraploid type *b* blastocysts lacked an ICM. Arrow indicates the ICM. Scale bar: 50 µm.

From a total of 931 *Oct4*-EGFP tetraploid blastocysts (day 3 after fusion), we found that 56% (519/931) of the expanded blastocysts have a distinct clump of GFP**^+^** ICM cells. These blastocysts, which we designated type *a* embryos, are morphologically similar to typical diploid blastocysts. On the other hand, 44% (412/931) of the tetraploid blastocysts we examined did not have an ICM, and were designated type *b*. Type *a* and type *b* embryos, can be readily distinguished by virtue of *Oct4*-EGFP fluorescence under the microscope (Fig. 1A and 1B). We then performed immunostaining of the type *a* and type *b* blastocysts with OCT4 (specific to ICM cells) and CDX2 (specific to trophoblast) antibodies; indeed, all the type *a* blastocysts had a layer of CDX2 positive cells with a small clump of OCT4 positive cells (≥4 OCT4 positive cells) in each embryo; while in type *b* blastocysts have CDX2 positive cells, but the OCT4 positive cells were completely absent, or were less than three cells scattered around the outer layer of the trophectoderm in the embryos (Fig. 1C).

**Figure 2:**
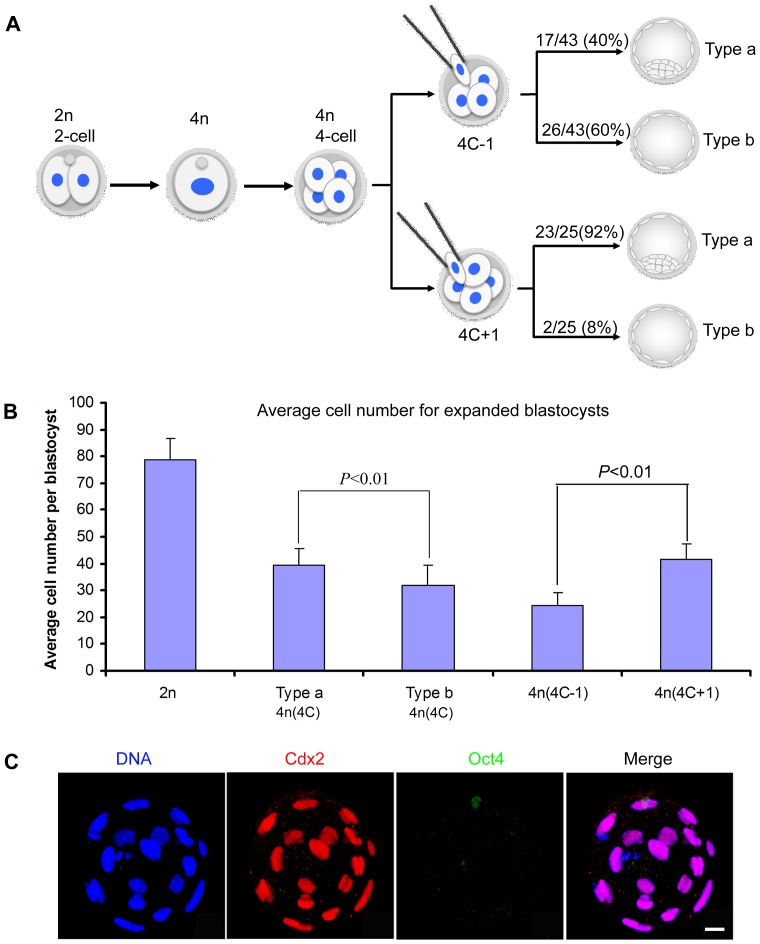
Cell number of early embryos determines the formation of type *a* and type *b* blastocysts. (A) Schematic illustration of how increasing or decreasing one blastomere at the 4-cell stage 4n embryos significantly affects type *a* and type *b* blastocyst formation. 4C-1: Removal of one blastomere at the 4-cell stage, the ratio of type *a* and type *b* blastocysts are 40% and 60% (*χ^2^*-test, *P*<0.01), respectively. 4C+1: Injection of an additional tetraploid blastomeres at the 4-cell stage, the ratio of type *a* and type *b* blastocysts are 92% and 8% (*χ^2^*-test, *P*<0.01), respectively. The ratio of type *a* and type *b* blastocysts in normal 4n embryos are 56% and 44%. (B) Average cell numbers of expanded blastocysts. 4n(4C):The average cell number in type *b* blastocysts from normal 4n embryos is lower than in type *a* blastocysts (*t*-test, *P*<0.01). 4n(4C-1): The cell number in the blastocysts from embryos with one blastomere removed at the 4-cell stage (type *a* and type *b* were not grouped). 4n(4C+1): The cell number in the blastocysts from embryos where one 4n blastomere was added at the 4-cells stage. 2n: Normal expanded 2n blastocysts. (C) A type *b* 4n blastocyst from 4C-1 embryos showing the decreased cell number and missing the ICM. Scale bar: 20 µm.

The average cell number (ACN) for type *a* and type *b* blastocysts was next examined. The ACN is significantly lower for type *b* (31.8±7.8) than type *a* blastocysts (39.2±6.2) (*t*-test, *P*<0.01), which is approximately half of that of diploid blastocysts of the same age (78.8±7.9) (Table 1 and Fig. 2A, B). To determine if the blastomere number at early embryonic stages is a factor that affects formation of an ICM during embryogenesis, we removed one blastomere from 4-cell stage 4n embryos (4C-1) or injected one additional tetraploid blastomere into 4-cell stage 4n embryos (4C+1). The percentages of type *a* and type *b* expanded blastocysts (on the 3^rd^ day after fusion), as well as ACNs were determined for this cohort of experimentally manipulated embryos (Fig. 2). In the 4C-1 embryos, the percentages of type *a* and type *b* were 40% and 60%, respectively; which were significantly different than for non manipulated 4n blastocysts (60% 4C-1 type *b* versus 44% 4C type *b* embryos, *χ* ^2^-test, *P*<0.01). On the other hand, the ACN was decreased to about 24 cells in 4C-1 type *b* blastocysts (24.9±4.7 for type *a* and 24.1±4.6 for type *b*; *t-*test, *P* = 0.337) (Fig. 2 and Table 1). On the contrary, in 4C+1 embryos, over 90% of the blastocysts were found to be the type *a* and had an ACN increased to 42.3±5.5 whereas the remaining type *b* had 35.3±1.7 cells (*t-*test, *P*<0.01) (Fig. 2 and Table 1). These results show that the number of blastomeres at the time of cavitation is a critical factor for the formation of an ICM in mouse blastocysts.

**Table 1:** The average cell number of expanded 4n blastocysts.

2n	4n(4C)		4n(4C+1)		4n(4C-1)	
	Type a	Type b	Type a	Type b	Type a	Type b
78.8±7.9(19)	39.2±6.2(40)	31.8±7.8(33)*	42.3±5.5(11)	35.3±1.7(4)*	24.9±4.7(10)	24.1±4.6(11)

*t-test, *P*<0.01, data was compared between type *a* and type *b* in each group; 4C+1: blastocysts produced by injection of one additional tetraploid blastomere at the 4-cell stage; 4C-1: blastocysts produced by removal of one tetraploid blastomere at the 4-cell stage.

Numbers in the parentheses are the embryos counted.

### Embryonic stem cell (ESC) lines can be readily derived from type *a* 4n blastocysts

**Figure 3:**
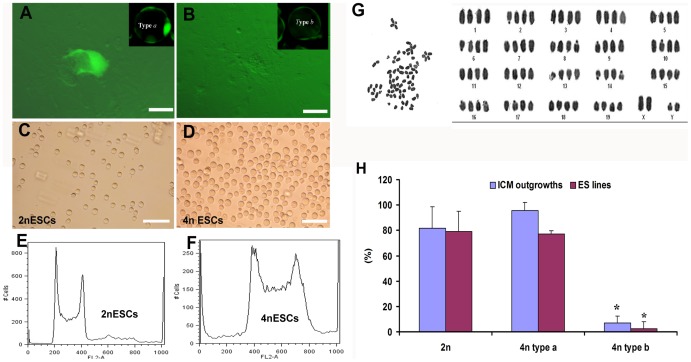
Embryonic stem cell (ESC) lines derived from type *a* 4n blastocysts. (A) Inner cell mass (ICM) outgrowth on MEFs from a type *a Oct4*-EGFP 4n blastocyst at the 4^th^ day of culture in ESDM. (B) An embryonic remnant from a type *b* 4n blastocyst at the 4^th^ day of culture in ESDM. (C, D) ESCs derived from both 2n embryos and type *a* 4n embryos. Note the larger cell size for 4nESCs (D) than 2nESCs (C). (E) Karyotype of 4nESCs (4n = 80). (F) Efficiency of ESC line derivation for 4n blastocysts from *Oct4*-EGFP mouse strain. The efficiency of ESC derivation for type *a* 4n blastocysts is similar to that for 2n blastocysts, while the efficiency for type *b* 4n blastocysts is significantly decreased (*P*<0.01). The type *a* and type *b* embryos were identified by visualization of the *Oct4*-EGFP under the fluorescence microscope.

We next determined if 4n blastocysts maintain the capacity to generate ESC lines. Blastocysts from *Oct4*-EGFP mice were grouped into type *a* and type *b* by visual inspection under a florescence microscope; each blastocyst was placed in individual wells of a 96-well plate, seeded with a mouse embryonic fibroblast (MEFs) feeder layer and freshly prepared ESC derivation medium (ESDM). Blastocysts were grown for 4–5 days without changing the medium. Within 3–4 days, the type *a* blastocysts were obviously bipartite with distinct ICM outgrowths and an outer ring of trophoblast cells, as well as a clump of *Oct4*-EGFP positive cells (Fig. 3A). By contrast, most of the type *b* blastocysts developed into a layer of cell remnants without *Oct4*-EGFP positive ICM outgrowths (Fig.3B and Table 2). ESCs derived from type *a* 4n blastocysts were morphologically larger in cell size than diploid ESCs (2.53±0.38 µm for 2nESCs and 3.84±4.2 µm for 4nESCs; *t*-test, *P*<0.01) (Fig. 3C and D); Karyotyping confirmed these ESCs as tetraploid or near tetraploid (70<n<90) (Fig.3E, F), although higher frequencies of aneuploidy (over 70%) in these ESCs were observed. From a total of 22 type *a* 4n blastocysts, we derived 17 ESC lines (77%), an efficiency similar to that of 2n blastocysts (78.9%) (Table 2). This result indicates that type *a* 4n blastocysts retain pluripotency as 2n embryos do. Conversely, approximately 95% of the type *b* blastocysts, identified by virtue of the lack of an Oct4-EGFP+ ICM, failed almost entirely to produce ESC lines (only 4.6% generated ESC lines, compared to 77.3% for type *a* blastocysts, *χ* ^2^-test, *P*<0.01) (Fig. 3H and Table 2). The 2 cell lines obtained from the type *b* blastocysts probably come from the OCT4+ cells occasionally found in the embryos. These data confirm that type *b* 4n blastocysts have no ICM and consequently have lost the ability to produce ES cells.

**Table 2:** The efficiency of ESC derivation for tetraploid blastocysts.

Embryos	No. blastocysts	No. ICM outgrowths (%)	No. ESC lines derived (%)
2n	38	31(81.6)	30(78.9)
Type a (4n)	22	21(95.5)	17(77.3)
Type b (4n)	43	9(20.9)*	2(4.7)*

*χ^2^-test, *P*<0.01. All the embryos were produced by crossing B6D2F1 females with *Oct4*-EGFP males, embryos were recovered at the 2-cell stage (42–46h post hCG injection).

### Tetraploid ESCs (4nESCs) are pluripotent and can contribute to chimeras

**Figure 4:**
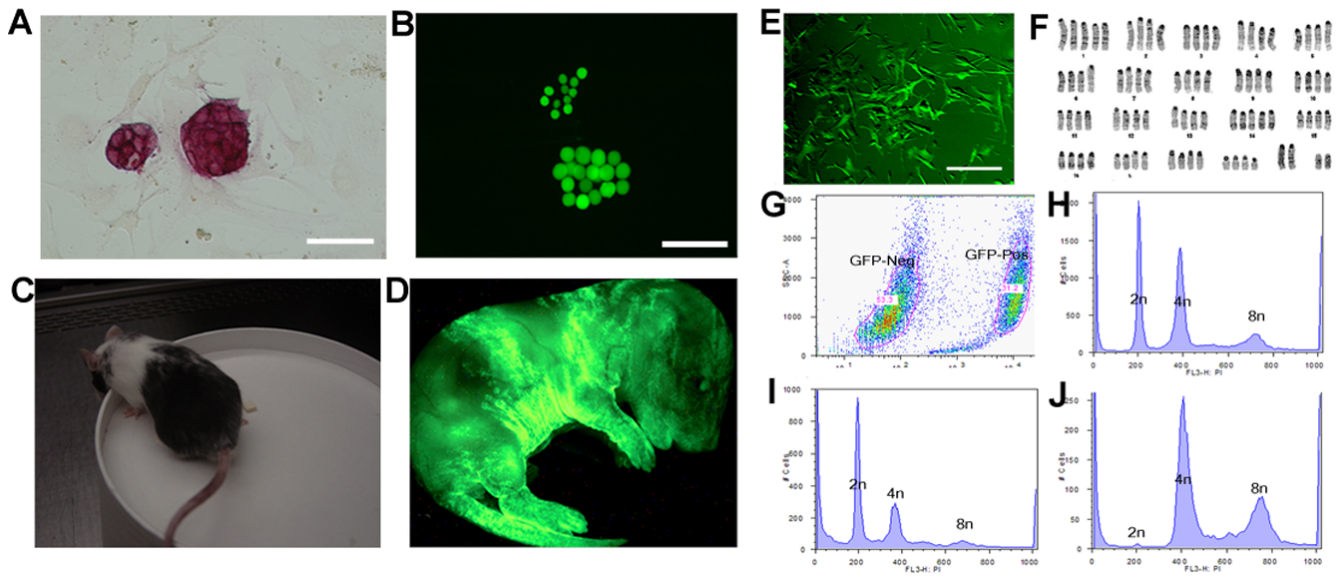
4nESCs are pluripotent and contribute substantially to chimeras. (A, B) 4nESCs express pluripotent markers, such as AP staining positive (A) and OCT4 expression (B). (C) A chimera (4n/2n) obtained by injection of 4nESCs (black coat color) into a 2n host blastocyst (white coat color). The chimera showed over 50% of 4nESCs contribution judging by the coat color. (D) A new born pup showed the contribution of 4nESCs (evidenced by the CMV promoter driven EGFP) in the 4n/2n chimera. (E) Fibroblasts from a newborn 4nESC/ICR chimera cultured *in vitro*, the derivatives of 4nESCs are EGFP^+^. (F) Karyotype of EGFP^+^ fibroblasts from chimera (4n = 82, (1, +1; 14, +1)). (G) Two populations of fibroblasts (EGFP^−^ and EGFP^+^). (H) The DNA content of the total population of fibroblasts stained with PI shows three peaks: 2n, 4n and 8n. (I) The DNA content of sorted EGFP^−^ fibroblasts shows two major peaks: 2n and 4n. (J) The DNA content of sorted GFP^+^ fibroblasts show two peaks: 4n and 8n. Scale bar: 200 µm.

To determine if the 4nESCs are pluripotent, we performed immunostaining and blastocyst injection for the generation of chimeras. The 4nESCs have alkaline phosphatase (AP) activity (Fig.4A) and express OCT4 (Fig.4B); 4nESCs are also immunoreactive for SSEA-1, but not for SSEA-3, SSEA-4, TRA-1-60, and TRA-1-81, similar to 2nESCs (data not shown). To test the pluripotency of 4nESCs *in vivo*, we injected the 4nESCs derived from *Oct4*-EGFP mice (black coat color) or 4nESCs constitutively expressing EGFP, into ICR (albino coat color) diploid blastocysts. The 4nESCs were found to contribute substantially to the 4n/2n chimeras (Fig.4C and D); we detected 4nESC-derived cells in almost all the organs and tissues (Data not shown). Fibroblasts from EGFP-4nESCs chimeras were further isolated and cultured *in vitro*, the EGFP^+^ cells were sorted and karyotyped and the 4nESC-derived cells were found to remain tetraploid or near tetraploid karyotype after *in vivo* differentiation, as expected (Fig. 4). Our results thus demonstrate that 4nESCs derived from type *a* blastocysts are pluripotent and can differentiate into most, if not all, tissue types in chimeras, suggesting that tetraploidy does not affect the developmental potential of ESCs in embryos.

### Completely ESC-derived mice obtained by tetraploid complementation using ICM-deficient blastocysts

**Figure 5:**
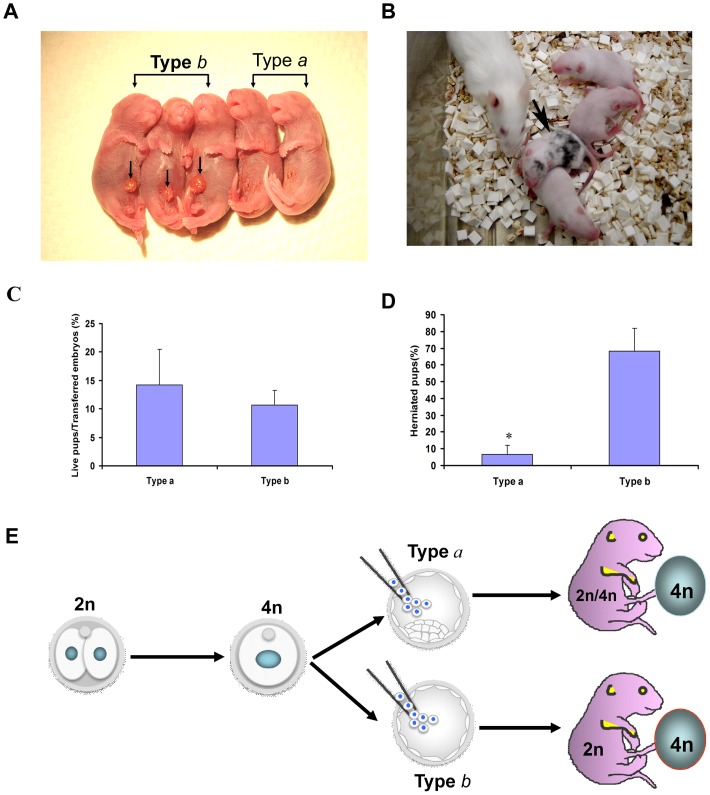
ESC mice produced by tetraploid complementation using both type *a* and type *b* 4n blastocysts. (A) Newborn pups obtained using type *a* and type *b* 4n blastocysts by tetraploid complementation. Pups from type *b* 4n blastocysts are frequently displaying abdominal hernia (arrow indicated), while pups from type *a* 4n blastocyst are all normal. (B) A litter of ESC pups (white coat color) produced using type *a* 4n blastocysts (black coat color); one pup (arrow indicated) displayed substantial contribution of cells from the host 4n embryo (over 20% of contribution from the host embryo judging by the coat color). (C) The efficiency to obtain ESC mice from type *a* or type *b* 4n blastocysts is not significantly different (*χ^2^*-test, *P* = 0.13). (D)The frequency of herniated pups using type *b* blastocysts is significantly higher than using type *a* blastocysts (*χ^2^*-test, *P*<0.01). (E) A model for tetraploid complementation illustrates ESC mice from type *a* 4n blastocysts are possibly 2n/4n chimeras, whereas ESC mice from type *b* 4n blastocysts could be pure ESC-derived.

To test the potential of type *a* and type *b* 4n blastocysts to make ESC mice by tetraploid complementation, we derived an ESC line from albino B6 (albino coat color), injected these ESCs (2n) into type *a* or type *b* 4n blastocysts (black coat color) and subsequently transferred the chimeric embryos separately into different surrogates. Of a total of 415 type *a* 4n blastocysts injected and transferred, 59 live ESC pups were obtained. Notably, over 10% of the type *a* 4n blastocyst-derived ESC mice were obviously chimeric (7 out of 59 pups), with detectable contribution of cells from the host tetraploid embryos as judged by coat color (Fig. 5B). Type *b* 4n blastocysts were also found to have the potential to generate ESC mice by tetraploid complementation; from a total of 384 type *b* 4n blastocysts injected with 2nESCs, 41 live ESC-derived pups were obtained (Fig. 5A, B and Table 3). The frequency of live pups born was not significantly different between type *a* and type *b* 4n blastocysts (14.2% versus 10.7%, *χ* ^2^-test, *P* = 0.13). As the type *b* 4n blastocysts have been depleted of the ICM, they effectively serve as a “trophoblastic vehicle” to produce ESC-derived mice. Therefore these embryos should not have any cell originating from the ‘host’, type *b* 4n blastocyst. As expected, none of these ESC mice derived from the type *b* 4n blastocysts were found to be chimeric judging by the coat color. Remarkably, about 70% of the type *b* 4n blastocyst-derived ESC pups displayed abdominal hernia, a frequency that is significantly higher than that of herniated pups derived from the type *a* 4n blastocysts (Fig. 5A and D, *χ* ^2^-test, *P*<0.01). Co-injection of 4nESCs and 2nESCs into the type *b* 4n blastocysts could partially improve the ratio to obtain normal pups (Table 3). The herniated pups, with no additional over gross morphological defects observed, could survive to adulthood. These results show that even though completely ESC-derived mice can be obtained from type *a* blastocysts, 4n cells from the host are not always excluded from the chimeric mouse. Conversely, the use of only ICM-depleted, type *b* blastocysts ensures the exclusion of 4n cells from the ESC-derived animal and a higher efficiency in the generation of completely ESC-derived mice.

**Table 3:** The efficiency to obtain ESC mice by tetraploid complementation.

Blastocysts	ESCs	No. embryos transferred	No. surrogates	No. live pups (%)	No. herniated pups (%)
Type *a*	2nESCs	415	20	59(14.2)	4(6.8)*
Type *b*	2nESCs	384	19	41(10.7)	28(68.3)
Type a	Co-injection 2nESCs 4nESCs	106	6	12(11.3)	0
Type b	Co-injection 2nESCs 4nESCs	52	3	5(9.6)	2(40.0)

*χ^2^-test, *P*<0.01.Percentage of live pups (%) = No. live pups/No. embryos* 100%; Percentage of herniated pups(%) = No. herniated pups/No. live pups*100%; Co-injection of 2nESCs and 4nESCs into tetraploid blastocysts, each blastocysts received at least ten 2nESCs and ten 4nESCs.

## Discussion

A typical mouse blastocyst consists of trophectoderm and inner cell mass (ICM). We show here that two types of mouse 4n blastocysts can be distinguished, which we classify as type *a* or type *b*, based on the presence or absence of ICM, assessed by EGFP expression from the Oct-4 promoter in a transgenic strain and by immunofluorescence on fixed samples. Type *a* 4n blastocysts are morphologically similar to normal diploid blastocysts that consist of both TE and ICM, while the type *b* 4n blastocysts lack an ICM. Given that 4n blastocysts have approximately half the number of cells than 2n blastocysts, the cell number at the time of embryo cavitation may be critical for the generation of an ICM. Consistently, our data show that the average cell number (ACN) for type *b* 4n blastocysts is lower than type *a* 4n blastocysts, and that increase (4C+1) or reduction (4C-1) in one blastomere at the 4-cell stage leads to a significant decrease or increase in the incidence of type *b* 4n blastocyst formation, respectively (Fig. 2A and B). This phenomenon is likely to be due to the topology and the position of cells in 4n embryos at the time of cavitation. It has been shown that cell density and position during blastocyst formation is translated via the Hippo pathway into lineage-specific gene expression [25], [26], [27], [28]. Inhibition of the Hippo cascade leads to exclusive expression of CDX2 in outside cells, whereas its activation in inner cells results in downregulation of CDX2 [28]. Mutual repression between CDX2 and OCT4 eventually result in restriction of their expression to the TE and ICM, respectively [29] and establishment of lineage identity. In our experiments, when diploid embryos are induced to become 4n by blastomere fusion, the cell number of embryos is decreased to half the number present in age-matched 2n embryos, however the timing of key embryonic events is not affected [30]. Consequently, all the blastomeres at the 8-cell stage will be positioned on the outside topologically in a 4n embryo, whereas a 2n embryo of the same age comprises 16-cells, and therefore, will have more cells inside of the embryo [31]. Tetraploid embryos, therefore, are likely not to have enough inner cells (if any) at the time of cavitation, thus giving rise to a high number of type *b*, 4n blastocysts, lacking an ICM.

To address whether tetraploid ES cells retain pluripotency and the ability to contribute to embryonic tissues, we derived and characterized ESC lines from type *a* blastocysts. These 4nESC lines show a typical ESC morphology, rapid growth, and the ability to remain undifferentiated in the absence of feeder cells but in the presence of LIF for more than 20 passages. However, chromosome spreads for 4nESCs revealed variable, often high frequencies of aneuploidy (>70%), thus indicating the genome of 4nESCs is very unstable in culture. This instability of the tetraploid genome might be caused by the acquisition of extra centrosomes that could compromise the assembly of a bipolar spindle [32]. Nevertheless, our results show that 4nESCs express all the pluripotency markers typical of 2n ES cells and can contribute to most of the tissues and organs in chimeras. Moreover, the derivatives of 4nESCs in chimeras remain tetraploid or near tetraploid after *in vivo* differentiation (Fig. 4), thus suggesting that tetraploidy does not affect the pluripotency of the 4nESCs. These data indicates that the use of type *a* 4n blastocysts for tetraploid complementation would not necessarily prevent the incorporation of host 4n cells to the embryo. Given this scenario, we examined whether these tetraploid embryos could contribute to ESC mice produced by tetraploid complementation. About 10% of the ESC mice obtained from type *a* 4n blastocysts were chimeric judging by the coat color. We can exclude the possibility of contamination of 2n embryos by carefully monitoring the presumed 4n embryos for the blastomere fusion and the first embryonic cleavage after fusion (embryos that become 2-cell within 3h after blastomeres fusion were not used for tetraploid complementation). We also confirmed that all the ESC lines derived from 4n embryos have a tetraploid karyotype. Thus, we conclude that the 10% of distinct chimeras derived from type *a* 4n blastocysts are 2n/4n chimeric. Therefore, our results show that tetraploid cells are not completely excluded from the mice produced by tetraploid complementation if type *a* 4n blastocysts are used, suggesting that tetraploidy is not itself the major factor for the biased segregation observed in 2n/4n chimeras.

ESC mice produced by injecting 2nESCs into 4n blastocysts exhibit a segregation of cells such that tetraploid cells rarely contribute to the embryo proper; these mice have traditionally been considered to be ‘completely’ ES cell-derived [8], [14], [15], [16], [17], [20], [33]. However, tetraploid cells have previously been detected in the body of 2n/4n chimeras, particularly with sensitive assays that provide single-cell resolution in early embryos and neonatal pups [5], [8], [20] and in this study. There is no doubt that mice produced by the tetraploid complementation method result in an extremely high contribution of ES cells to the resulting mice. However there is still no conclusive evidence that any of these mice are completely ES cell-derived. It is relatively straightforward to prove that a mouse is chimeric by providing evidence of the presence of tetraploid cells - just one tetraploid cell is sufficient. By contrast, it is difficult, if not impossible, to prove that a mouse is not chimeric by demonstrating the absence of tetraploid cells. Since this analysis would involve the processing of all tissues and cells in the mouse, it would not be practical to routinely screen every all-ESC mouse generated by tetraploid complementation if they are to be bred to adulthood. We show here that the type *b* 4n blastocysts are missing the ICM and thus the pluripotent epiblast compartment, and consequently have very low capacity to derive ESC lines; accordingly, the type *b* 4n blastocysts should not make any contributions to the fetus of chimeric mice obtained from these embryos. Therefore, the use of ICM-deficient, type *b*, 4n blastocysts provides an alternative method to routinely generate all-ESC derived mice with the confidence that no ICM cells from the host 4n embryo will contribute to the animal. Notably, these complete ESC mice obtained from type *b* 4n blastocysts display high frequency of abdominal hernia in the absence of any other over morphological or behavioral defects. Indeed, this phenotype was noted in animals generated from various ESC lines from different backgrounds (data not shown), and was almost exclusively found with the type *b* 4n blastocysts. When co-injectiing 4nESCs and 2nESCs into type *b* 4n blastocysts, a partially rescue of the ratio of herniated pups was observed, suggesting that the presence of hernia in the complete ESC mice may be due to the lower cell number that the type *b* 4n blastocysts have. Collectively, our data provide the evidence that the segregation of 4n blastocysts into type *a* and type *b* is the major mechanism to produce completely ES cell-derived mice by tetraploid complementation via blastocyst injection.

It has been previously shown that the primitive endoderm (PrE) derivatives in tetraploid complementation assays are derived from the 4n host embryo [12], consistently with the low efficiency of ESCs contribution to this lineage in chimeras [34], [35]. Therefore, the origin of the PrE in chimeric embryos made with host type *b* 4n blastocysts remains unclear at this stage. One possibility is that the few OCT4+ cells that are occasionally found in type *b* blastocysts (<3 cells) give rise to the PrE in the chimeric embryo. The low frequency of survival of this kind of chimeras (about 10% embryos develop to term in this study) would agree with this hypothesis. Alternatively, some of the ESCs injected in the embryo may be forced in this scenario to differentiate into PrE. This behavior has not been previously observed, however, recent findings that subpopulations of totipotent ESCs are found in culture [35], [36] suggest this could be the case, at least in a number of chimeras. Lastly, albeit unlikely, tetraploid cells initially positioned on the trophoblast of the host embryo, might be forced to change position and contribute to the PrE in these embryos. Although it is currently unclear how the PrE lineage is generated in these embryos, whether any (or several) of the above alternatives can provide a mechanism will be addressed in the future study.

In conclusion, we show that tetraploid blastocysts can be classified into two types, which we refer to as type *a* and type *b*, based on the presence or absence of an ICM. When the type *a* 4n blastocysts are used for blastocyst injection, the resulting ESC mice are often 2n/4n chimeric; however, when the type *b* 4n blastocysts are used, the resulting ESC mice are completely ESC-derived (Fig. 5E). Our results therefore provide evidence that completely ESC-derived mice can be possible obtained through tetraploid complementation if type *b* 4n blastocysts are used, and shed light on the mechanism(s) of tetraploid complementation.
